# Sex differences in the association of BMI and weight perception with depression and suicidality among Korean adolescents

**DOI:** 10.1371/journal.pone.0328549

**Published:** 2025-08-06

**Authors:** Eunha Jeong

**Affiliations:** School Health Clinic, Doonchon Middle School, Seoul, Republic of Korea; Trinium Woman's Hospital, KOREA, REPUBLIC OF

## Abstract

This study aimed to investigate sex-based differences in the effects of weight perception on depression and suicidality in Korean adolescents with and without obesity. A multiple logistic regression analysis stratified by sex was conducted using the 2023 Korea Youth Risk Behavior Survey data of 51,462 middle and high school students. BMI-based obese adolescents comprised a higher proportion of male participants (22.5%) than female participants (17.4%). The rate of underweight perception was higher among male participants (32.4%) than among female participants (23.3%); however, the rate of overweight perception was higher among female participants (37.3%) than among male participants (36.0%). The risk of depression or suicidality was higher among female participants (30.7% and 18.2%, respectively) than among male participants (21.3% and 10.7%, respectively). In female participants without obesity, the risk of depression and suicidality with overweight perception increased by 1.106 times (95% confidence interval [CI] 1.020–1.199) and 1.295 times (95% CI 1.176–1.427), respectively, compared to that with underweight perception as the reference group. However, this difference was not statistically significant among male participants without obesity. The findings of this study suggest that to improve the mental health of adolescents, proper weight recognition education along with obesity prevention should be implemented, and sex-specific interventions should be considered.

## Introduction

Globally, one in seven adolescents aged 10–19 years experiences mental health problems. If left unaddressed, these conditions may persist into adulthood, impairing both physical and mental health and reducing opportunities to achieve a better quality of life [1]. South Korea has the highest suicide rate among individuals aged 20 and older in the Organisation for Economic Co-operation and Development (OECD) member countries. Among youths aged 10–24 years, the OECD average suicide rate is 5.9 per 100,000, whereas Korea ranks second with a rate of 12.4 per 100,000, more than twice the OECD average [2]. Adolescence is a critical transitional period into adulthood [1], during which predispositions to mental health problems may emerge or intensify, potentially leading to suicidal behavior. As of 2022, suicide was the leading cause of death among Korean adolescents, accounting for 42.3% of all deaths. Its prevalence was more than three times higher than that of cancer (12.1%), which ranked second [2]. According to the 2024 White Paper on Suicide Prevention [2], the most frequently cited cause of adolescent suicide is psychological distress. Therefore, it is essential to examine suicide in close association with depression.

Depression is the leading cause of illness and disability among adolescents [1] and a major contributing factor to suicide [3]. Its prevalence has increased significantly in recent years [4], with a further increase observed in Korea following the COVID-19 pandemic [5]. Depression impairs school attendance and academic performance, intensifies social withdrawal, loneliness, and isolation [1], and increases the risk of suicide [3]. Therefore, addressing both depression and suicide should be a top priority in efforts to improve adolescent mental health.

The global prevalence of overweight and obesity among children and adolescents aged 5–19 years has increased sharply, rising from 8% in 1990 to 20% in 2022. Adolescent obesity has, in fact, quadrupled during this period [6]. A multinational study involving adolescents aged 13–17 years across 20 countries reported an overweight or obesity rate of 18.2% in the mid-2010s [7]. In Korea, as of 2023, 21% of adolescents—from the first year of middle school to the third year of high school—were classified as overweight or obese [8]. It is well established that being overweight or obese increases the risk of both immediate and long-term health problems. Adolescents with excess weight are more likely to develop severe obesity and noncommunicable diseases such as diabetes and cardio-cerebrovascular conditions [9]. They are also at heightened risk for psychosocial challenges. Obesity-related stigma and discrimination can have far-reaching negative effects on academic performance and overall quality of life [6].

Previous studies have consistently demonstrated a strong association between obesity and mental health problems, particularly depression and suicidal tendencies [10–12]. However, research focusing solely on the direct link between obesity and mental health may overlook critical underlying mechanisms, especially among adolescents. Adolescence is a critical stage of development, characterized by rapid physical changes [13] and increased attention to appearance [14]. During this period, being overweight or obese is closely linked to adolescents’ perceptions of their body image [13]. Body image is a multifaceted concept that encompasses individuals’ subjective perceptions and attitudes toward their own bodies [15]. IN 2023, 21.7% of Korean adolescents reported body image distortion—believing they were overweight or obese despite being underweight or of normal weight—a slight increase from the previous year [8]. Moreover, appearance has been reported as the most distressing concern among Korean teenagers [16]. Given prior evidence that distorted weight perception adversely affects adolescent mental health, including depression and suicidality [14,17–19], it is essential to investigate how both actual weight status and perceived weight influence psychological well-being.

To date, most studies on adolescent suicide have either focused exclusively on suicidal thoughts [18,20] or have examined suicidal thoughts, plans, and attempts as separate outcomes [10,17,21]. However, suicide is better understood as a behavioral continuum, ranging from ideation to planning and eventual attempts [22]. Accordingly, the present study aimed to clarify the relationship between weight perception and suicidality among adolescents with and without obesity, using an integrated definition of suicidality encompassing suicidal thoughts, plans, and attempts [5]. In addition, numerous studies have reported sex-based differences in the factors associated with obesity, weight perception, depression, and suicidality [5,17,18,20,21].

The present study aimed to investigate sex-based differences in the effects of weight perception on depression and suicidality among Korean adolescents, stratified by obesity status. It also sought to provide practical evidence to inform sex-specific intervention strategies for adolescent depression and suicide prevention.

## Materials and methods

### Study design and participants

This study was a secondary data analysis using data from the 19th (2023) Korea Youth Risk Behavior Survey (KYRBS). The KYRBS is a nationwide cross-sectional study conducted annually among students from the first year of middle school (age 12) to the third year of high school (age 18) to understand the current status and trends of adolescent health behavior. The target population of the 19th KYRBS was middle and high school students in Korea as of April 2023. Samples were extracted through population stratification, sample distribution, and sampling stages. An anonymous, self-reported, online survey was conducted with 56,935 students from 800 schools. Among them, 52,880 students from 799 schools participated in the survey, representing a 92.9% participation rate. After conducting an exploratory data analysis on the main variables and covariates, data from 51,462 respondents were used for the final analysis, after excluding 1,418 respondents with missing values. The mean age of the participants was 15.08 years (SD = 1.74) for male adolescents and 15.07 years (SD = 1.73) for female adolescents. This study was approved by the Public Institutional Review Board of the Ministry of Health and Welfare (IRB No. P01-202409-01-023). All procedures performed in this study complied with the ethical standards of this Board and with the Declaration of Helsinki. The requirement of informed consent was waived as the study used secondary data.

### Measures

Sex (male/female) was employed as the primary explanatory variable.

#### Weight-related variables: BMI and weight perception.

Body mass index (BMI) was calculated by dividing weight (kg) by the square of height (m). Based on the BMI percentile for age and sex presented in the 2017 Korean National Growth Charts, < 5 percentile was defined as underweight, ≥ 5 percentile and < 85 percentile as normal weight, ≥ 85 percentile and < 95 percentile as overweight, and ≥ 95 percentile as obesity [23]. Ultimately, participants in the underweight and normal weight category were classified as non-obese, whereas the others were classified as obese.

Weight perception was assessed using a single question, “What do you think your body shape is?,” and responses was reclassified into underweight (very thin, slightly thin), normal weight (normal), and overweight (slightly fat, very fat) perceptions. This item, derived from the KYRBS, a nationally standardized surveillance system, has been widely used in previous studies as a valid indicator of adolescents’ subjective weight perception [18,20].

#### Mental health-related variables: depression and suicidality.

Depression was assessed using a single binary-response item from the KYRBS: “In the past 12 months, have you felt sad or hopeless enough to stop your daily life for two weeks in a row?” This item has been commonly used in large-scale adolescent mental health studies and national surveillance systems, including previous KYRBS-based studies [18,20].

Suicidality is a concept that encompasses suicidal thoughts, plans, and attempts [5]. In line with recent frameworks that conceptualize suicidality as a behavioral spectrum, this study used a composite measure including suicidal thoughts, plans, and attempts. These three standardized items from the KYRBS have been previously utilized in adolescent mental health research [18,20], offering a comprehensive understanding of suicidality [5,22]. It was assessed based on three questions, “In the last 12 months, have you seriously considered suicide?,” “In the last 12 months, have you made any specific plans to commit suicide?,” and “In the last 12 months, have you attempted suicide?” Participants were classified as having suicidality if they answered yes to any of these questions; otherwise, they were classified as not having suicidality.

#### Covariates.

Covariates were selected based on previous studies examining factors related to depression and suicidality [5]. These included school level (middle school or high school), school location (urban or rural), economic status (high, middle-high, middle, middle-low, or low), academic performance (high, middle-high, middle, middle-low, or low), highest education level of parents (high school or lower, college or higher, or unknown), current smoking status (no or yes), and current drinking status (no or yes). Based on the classification criteria for school locations used by Woo, et al. [5], Seoul, Busan, Daegu, Incheon, Gwangju, Daejeon, Ulsan, and Sejong were classified as urban, whereas Gyeonggi, Gangwon, Chungbuk, Chungnam, Gyeongbuk, Gyeongnam, Jeonbuk, Jeonnam, and Jeju were classified as rural. In addition, the highest education level the father or mother was regarded as the level of parental education, classified as high school or lower, college or higher, or unknown.

### Data analyses

All data were analyzed using the SPSS V29.0 program (IBM Institute, NY, USA) following the guidelines for complex sampling design provided by the Korea Disease Control and Prevention Agency. Values of P < 0.05 was considered statistically significant. First, a descriptive statistical analysis was performed to understand the characteristics of all variables. Second, the chi-square test was used to examine the differences in participants’ characteristics according to depression and suicidality. Third, multiple logistic regression analysis was used to explore the association between the combination of BMI and weight perception and depression or suicidality. To identify sex-based differences at each step, the analysis was subdivided for male and female participants.

## Results

### Characteristics of study participants and main variables

The characteristics of participants and major variables according to sex are presented in Table 1.

**Table 1:** Characteristics of the participants (N = 51,462).

Variables	Categories	Males participants (*N* = 26,120)	Females participants (*N* = 25,342)
		*n* (%)	*n* (%)
School level	Middle school	14,063 (53.8)	13,642 (53.8)
	High school	12,057 (46.2)	11,700 (46.2)
School location	Urban	11,119 (42.6)	11,117 (43.9)
	Rural	15,001 (57.4)	14,225 (56.1)
Economic status	High	3,357 (12.8)	2,744 (10.8)
	Middle-high	8,191 (31.4)	7,605 (30.0)
	Middle	11,571 (44.3)	11,830 (46.7)
	Middle-low	2,483 (9.5)	2,731 (10.8)
	Low	518 (2.0)	432 (1.7)
Academic performance	High	3,677 (14.1)	2,971 (11.7)
	Middle-high	6,317 (24.2)	6,685 (26.4)
	Middle	7,640 (29.2)	7,550 (29.8)
	Middle-low	5,906 (22.6)	6,078 (24.0)
	Low	2,580 (9.9)	2,058 (8.1)
Highest parental education level	≤ High school	3,250 (12.4)	3,895 (15.4)
	≥ College	14,316 (54.8)	15,962 (63.0)
	Unknown	8,554 (32.7)	5,485 (21.6)
Current smoking	No	24,719 (94.6)	24,649 (97.3)
	Yes	1,401 (5.4)	693 (2.7)
Current drinking	No	22,839 (87.4)	23,083 (91.1)
	Yes	3,281 (12.6)	2,259 (8.9)
BMI category	Obese	6,659 (25.5)	4,411 (17.4)
	Non-obese	19,461 (74.5)	20,931 (82.6)
Weight perception	Underweight	8,475 (32.4)	5,906 (23.3)
	Normal weight	8,248 (31.6)	9,984 (39.4)
	Overweight	9,397 (36.0)	9,452 (37.3)
Depression	No	20,559 (78.7)	17,553 (69.3)
	Yes	5,561 (21.3)	7,789 (30.7)
Suicidality	None	23,320 (89.3)	20,760 (81.9)
	Any	2,800 (10.7)	4,583 (18.1)

*Note*. BMI = body mass index

BMI-based obese adolescents comprised a higher proportion of male participants (22.5%) than that of female participants (17.4%). The rate of underweight perception was higher among male participants (32.4%) than among female participants (23.3%); however, the rate of overweight perception was higher among female participants (37.3%) than among male participants (36.0%).

The risk of depression or suicidality was higher among female participants (30.7% and 18.2%, respectively) than among male participants (21.3% and 10.7%, respectively).

### Differences in participants’ characteristics according to mental health

Table 2 shows the sex-based differences in participants’ characteristics according to depression.

**Table 2:** Sex-based differences in the characteristics of participants with and without depression (N = 51,462).

Variables	Males (*N* = 26,120)			Females (*N* = 25,342)		
	Depression		*p*	Depression		*p*
	No (*N* = 20,559) *n* (%)	Yes (*N* = 5,561) *n* (%)		No (*N* = 17,553) *n* (%)	Yes (*N* = 7,789) *n* (%)	
School level						
Middle school	11,071 (53.8)	2,992 (53.8)	.951	9,369 (53.4)	4,273 (54.9)	.029
High school	9,488 (46.2)	2,569 (46.2)		8,184 (46.6)	3,516 (45.1)	
School location						
Urban	8,704 (42.3)	2,415 (43.4)	.114	7,782 (44.3)	3,335 (42.8)	.025
Rural	11,855 (57.7)	3,146 (56.6)		9,771 (55.7)	4,454 (57.2)	
Economic status						
High	2,673 (13.0)	684 (12.3)	<.001	1,918 (10.9)	826 (10.6)	<.001
Middle-high	6,521 (31.7)	1,670 (30.0)		2,154 (31.1)	2,154 (27.7)	
Middle	9,265 (45.1)	2,306 (41.5)		8,356 (47.6)	3,474 (44.6)	
Middle-low	1,783 (8.7)	700 (12.6)		1,628 (9.3)	1,103 (14.2)	
Low	317 (1.5)	201 (3.6)		200 (1.1)	232 (3.0)	
Academic performance						
High	2,989 (14.5)	688 (12.4)	<.001	2,226 (12.7)	745 (9.6)	<.001
Middle-high	5,035 (24.5)	1,282 (23.1)		4,843 (27.6)	1,842 (23.6)	
Middle	6,134 (29.8)	1,506 (27.1)		5,382 (30.7)	2,168 (27.8)	
Middle-low	4,548 (22.1)	1,358 (24.4)		3,920 (22.3)	2,158 (27.7)	
Low	1,853 (9.0)	727 (13.1)		1,182 (6.7)	876 (11.2)	
Highest parental education level						
≤ High school	2,576 (12.5)	674 (12.1)	.179	2,622 (14.9)	1,273 (16.3)	.006
≥ College	11,207 (54.5)	3,109 (55.9)		11,154 (63.5)	4,808 (61.7)	
Unknown	6,776 (33.0)	1,778 (32.0)		3,777 (21.5)	1,708 (21.9)	
Current smoking						
No	19,676 (95.7)	5,043 (90.7)	<.001	17,298 (98.5)	7,351 (94.4)	<.001
Yes	883 (4.3)	518 (9.3)		255 (1.5)	438 (5.6)	
Current drinking						
No	18,343 (89.2)	4,496 (80.8)	<.001	16,376 (93.3)	6,707 (86.1)	<.001
Yes	2,216 (10.8)	1,065 (19.2)		1,177 (6.7)	1,082 (13.9)	
BMI category						
Obese	5,239 (25.5)	1,420 (25.5)	.937	3,029 (17.3)	1,382 (17.7)	.346
Non-obese	15,320 (74.5)	4,141 (74.5)		14,524 (82.7)	6,407 (82.3)	
Weight perception						
Underweight	6,573 (32.0)	1,902 (32.4)	<.001	4,058 (23.1)	1,848 (23.7)	<.001
Normal weight	6,624 (32.2)	1,624 (29.2)		7,183 (40.9)	2,801 (36.0)	
Overweight	7,362 (35.8)	2,035 (36.6)		6,312 (36.0)	3,140 (40.3)	

*Note.* BMI = body mass index.

Among male participants, significant differences in the presence or absence of depression were observed according to economic status, academic performance, parental education level, current smoking status, current drinking status, and weight perception. In contrast, among female participants, significant differences in the presence or absence of depression were observed according to school level, school location, economic status, academic performance, parental education level, current smoking status, current drinking status, and weight perception.

Table 3 shows the sex-based differences in participants’ characteristics according to suicidality.

**Table 3:** Sex-based differences in the characteristics of participants with and without suicidality (N = 51,462).

Variables	Males (*N* = 26,120)			Females (*N* = 25,342)		
	Suicidality		*p*	Suicidality		*p*
	No (*N* = 23,320) *n* (%)	Yes (*N* = 2,800) *n* (%)		No (N = 20,760) *n* (%)	Yes (N = 4,582) *n* (%)	
School level						
Middle school	12,448 (53.4)	1,615 (57.7)	<.001	10,864 (52.3)	2,778 (60.6)	<.001
High school	10,872 (46.6)	1,185 (42.3)		9,896 (47.7)	1,804 (39.4)	
School location						
Urban	9,957 (42.7)	1,162 (41.5)	.226	9,152 (44.1)	1,965 (42.9)	.139
Rural	13,363 (57.3)	1,638 (58.5)		11,608 (55.9)	2,617 (57.1)	
Economic status						
High	3,006 (12.9)	351 (12.5)	<.001	2,290 (11.0)	454 (9.9)	<.001
Middle-high	7,406 (31.8)	785 (28.0)		6,385 (30.8)	1,220 (26.6)	
Middle	10,441 (44.8)	1,130 (40.4)		9,829 (47.3)	2,001 (43.7)	
Middle-low	2,084 (8.9)	399 (14.2)		1,985 (9.6)	746 (16.3)	
Low	383 (1.6)	135 (4.8)		271 (1.3)	161 (3.5)	
Academic performance						
High	3,323 (14.2)	354 (12.6)	<.001	2,468 (11.9)	503 (11.0)	<.001
Middle-high	5,664 (24.3)	653 (23.3)		5,612 (27.0)	1,073 (23.4)	
Middle	6,920 (29.7)	720 (25.7)		6,363 (30.7)	1,187 (25.9)	
Middle-low	5,223 (22.4)	683 (24.4)		4,831 (23.3)	1,247 (27.2)	
Low	2,190 (9.4)	390 (13.9)		1,486 (7.2)	572 (12.5)	
Highest parental education level						
≤ High school	2,920 (12.5)	330 (11.8)	.504	3,142 (15.1)	753 (16.4)	.035
≥ College	12,778 (54.9)	1,538 (54.9)		13,079 (63.0)	2,883 (62.9)	
Unknown	7,622 (32.7)	932 (33.3)		4,539 (21.9)	946 (20.6)	
Current smoking						
No	22,215 (95.3)	2,504 (89.4)	<.001	20,392 (98.2)	4,257 (92.9)	<.001
Yes	1,105 (4.7)	296 (10.6)		368 (1.8)	325 (7.1)	
Current drinking						
No	20,615 (88.4)	2,224 (79.4)	<.001	19,190 (92.4)	3,893 (85.0)	<.001
Yes	2,705 (11.6)	576 (20.6)		1,570 (7.6)	689 (15.0)	
BMI category						
Obese	5,885 (25.2)	774 (27.6)	.006	3,455 (16.6)	956 (20.9)	<.001
Non-obese	17,435 (74.8)	2,026 (72.4)		17,305 (83.4)	3,626 (79.1)	
Weight perception						
Underweight	7,546 (32.4)	929 (33.2)	<.001	4,873 (23.5)	1,033 (22.5)	<.001
Normal weight	7,462 (32.0)	786 (28.1)		8,516 (41.0)	1,468 (32.0)	
Overweight	8,312 (35.6)	1,085 (38.8)		7,371 (35.5)	2,081 (45.4)	

*Note.* BMI = body mass index

Among male participants, significant differences in the presence or absence of suicidality were observed according to school level, economic status, academic performance, current smoking status, current drinking status, BMI, and weight perception. In contrast, among female participants, significant differences were observed in the presence or absence of suicidality according to school level, economic status, academic performance, parental education level, current smoking status, current drinking status, BMI, and weight perception.

### Odds ratio of mental health according to BMI and weight perception

Table 4 shows odds ratio of depression and suicidality according to BMI and weight perception stratified by sex.

**Table 4:** Adjusted OR (95% CI) for mental health based on BMI and weight perception categories stratified by sex (N = 51,462).

Depression	Male		Female	
	Obese	Non-obese	Obese	Non-obese
Weight perception				
Underweight	1	1	1	1
Normal weight	0.708 (0.341-1.468)	**0.853** **(0.790-0.922)**	**0.250** **(0.068-0.916)**	**0.858 (0.798-0.922)**
Overweight	0.722 (0.360-1.452)	1.002 (0.908-1.106)	**0.247** **(0.070-0.873)**	**1.106** **(1.020-1.199)**
Suicidality	Male		Female	
	Obese	Non-obese	Obese	Non-obese
Weight perception				
Underweight	1	1	1	1
Normal weight	**0.427** **(0.193-0.944)**	**0.871** **(0.785-0.966)**	**0.165** **(0.046-0.598)**	**0.832** **(0.761-0.910)**
Overweight	**0.432** **(0.205-0.909)**	1.079 (0.948-1.228)	**0.213** **(0.062-0.730)**	**1.295** **(1.176-1.427)**

*Note.* OR = odds ratio; CI = confidence interval; BMI = body mass index; Bold text represents significant values. Adjusted for school level, school location, economic status, academic performance, highest parental education level, current smoking status, and current drinking status.

Among male adolescents with obesity, weight perception was not identified as a risk factor for depression; however, among female adolescents, the odds ratio of depression decreased by 0.250 times (95% confidence interval [CI] 0.068–0.916) for normal weight perception and 0.247 times (95% CI 0.070–0.873) for overweight perception compared to that for underweight perception, which served as the reference group.

In contrast, among male adolescents without obesity, the odds ratio of depression decreased by 0.853 times (95% CI 0.790–0.922) for normal weight perception compared to that for the underweight perception. Among female adolescents without obesity, the odds ratio of depression decreased by 0.858 times (95% CI 0.798–0.922) for normal weight perception compared to the underweight perception. However, among female adolescents without obesity, the odds ratio of depression increased by 1.106 times (95% CI 1.020–1.199) for overweight perception compared to that for underweight perception.

Among male adolescents with obesity, the odds ratio of suicidality decreased by 0.427 times (95% CI 0.193–0.944) for normal weight perception and 0.432 times (95% CI 0.205–0.909) for overweight perception compared to that for underweight perception. Among female adolescents with obesity, the odds ratio of suicidality decreased by 0.165 times (95% CI 0.046–0.598) for normal weight perception and 0.213 times (95% CI 0.062–0.730) for overweight perception compared to that for underweight perception.

In contrast, among male adolescents without obesity, the odds ratio of suicidality decreased by 0.871 times (95% CI 0.785–0.966) for normal weight perception compared to that for underweight perception. Among female adolescents without obesity, the odds ratio of suicidality decreased by 0.832 times (95% CI 0.761–0.910) for normal weight perception compared to that for underweight perception; however, it increased by 1.295 times (95% CI 1.176–1.427) for overweight perception.

## Discussion

The present study revealed that sex-based differences were evident in terms of obesity, weight perception, depression, and suicidal tendencies among Korean adolescents.

Our findings align with previous research showing that adolescents face increasing risks of both obesity and mental health problems, including depression and suicidality. For example, national trends in South Korea demonstrate that obesity rates among adolescents have doubled over the past decade, especially among male adolescents [8,20]. Simultaneously, the prevalence of depression and suicidality has risen significantly, with female adolescents exhibiting greater overall vulnerability [4,5]. Although the prevalence of these mental health issues is generally higher among female adolescents, increasing attention is being paid to psychosocial risks among males as well [3].

In this context, our study highlights the complex associations between actual weight status, weight perception, and psychological outcomes. Among adolescents without obesity, those who perceived themselves as overweight were at increased risk of both depression and suicidality, an association that was particularly pronounced among females. In contrast, among male adolescents without obesity, weight perception was not significantly associated with either depression or suicidality. These sex-based differences suggest that body image and self-perception, rather than objective weight status alone, are critical in understanding adolescent mental health and developing targeted interventions.

Obesity is influenced by multidimensional factors, including physiological factors, such as sex hormones and genes, as well as lifestyle, such as food preferences and physical activity, or sociocultural factors [24]. In particular, after puberty, adolescents experience rapid changes in fat distribution and body composition due to the influence of sex hormones [24] and are influenced by sociocultural ideal body stereotypes and weight stigma, often shaped by sex-specific double standards. The mesomorph body type for male individuals and thin body type for female individuals are considered ideal body types, and both male and female adolescents internalize these standards without critical thinking [25]. A total of 81.8% of adolescents with obesity reported experiencing weight-related stigma, with female individuals being 2.6 times more likely to encounter such stigma than male individuals [26].

Unlike actual weight status, differences were found in weight perception. The rate of underweight perception was higher among male participants than among female participants and that of overweight perception was higher among female participants than among male participants. These findings align with those of previous studies, which reported that men underestimate their weight and women overestimate their weight [18,21,27,28]. Kim [29] stated that sex and age are the most critical causal factors related to weight evaluation and pointed out that young female individuals overestimate their weight due to heightened sensitivity to their appearance and social expectations regarding their physique. Recent findings [25] also indicate that this weight misperception is not limited to young female individuals, and young male individuals underestimate weight for the same reason. Interestingly, these trends in weight perception are observed across both eastern and western countries [21,27,29,30]. However, it is interesting to note that although Korea’s obesity rate is not high compared to the western countries [31], weight perception closely aligns with the Westernized typicality.

Sources of pressure regarding weight or body shape include society, parents, schools, peers, and mass media [24,26]. For example, parents are more likely to underestimate their children’s weight if their children are overweight or obese, and the risk of underestimating their children’s weight is higher for sons than for daughters [32]. Li, et al. [33] reported that underweight recognition by parents or grandparents is the most powerful predictor of overweight or obesity in children, with boys being more prone than girls to having their weight underestimated. Additionally, recent studies have reported that the discrepancy between objective and subjective weight status, caused by the internalization of unrealistic body ideals through media, increases the likelihood of body dissatisfaction or eating disorders [34,35]. The influence of media ideal internalization through negative physical self on social appearance anxiety is greater in girls than in boys [36]. In conclusion, a sex-based difference exists in the intensity of pressure on weight or body shape, with the acceptable range for male individuals being broader than that for female individuals.

This study showed that the rate of depression is higher among female participants than among male participants, supporting previous research findings [4]. The frequency or severity of depressive symptoms was also higher in girls than in boys [5]. In general, the sex-related gap in depression begins to appear in early adolescence, with several factors contributing to girls’ higher vulnerability to depression. These factors include (1) biological vulnerabilities such as genes, puberty timing, and puberty hormones; (2) affective vulnerability such as temperament; and (3) cognitive vulnerability such as rumination, objectified body consciousness, and a negative cognitive style. In other words, stress or negative life events play an important role in sex-based differences in biological, physical, and psychological changes accompanying puberty, along with the contribution of sociocultural factors such as gender inequality [37].

The prevalence of suicidality was higher among female participants than among male participants. Since the inception of the KYRBS in 2005, female participants have consistently exhibited higher suicidality rates than those of male participants [5]. These results are similar to those of previous studies conducted abroad [38,39]. According to a statistical analysis report on the causes of death, a sex-based gap was noted in the number of suicides that led to death. In 2016, 455 boys (61.2%) and 289 (38.8%) girls committed suicide, showing a large difference in the numbers between them; however, in 2019, the numbers had nearly equalized to 455 (51.9%) and 421 (48.1%) in boys and girls, respectively. Notably, the number of suicides among female adolescents has increased significantly in recent years [40]. Miranda-Mendizabal, et al. [39] identified eating disorders, dating violence, depression symptoms, and interpersonal problems as risk factors for suicidality among female individuals and conduct disorders and peer suicidality as risk factors for suicidality among male individuals. To fully understand the causes of mental health problems, a multidimensional approach, including genetic, biological, psychological, emotional, cognitive, and behavioral factors, as well as interaction experiences with the environment to which the individual belongs, is necessary [40]. Hence, follow-up studies should be aim to specifically identify the mechanism and the risk factors affecting sex-based differences in suicidality to prevent suicide in adolescents.

Another major finding of the present study was that, unlike male adolescents without obesity, in female adolescents without obesity, weight overestimation was as risk factor for poor mental health. Regardless of BMI, an association exists between overweight perception and mental health deterioration [17]. Furthermore, social body comparison or internalization plays a more decisive role than BMI in female adolescents’ body satisfaction and body image concerns [13]. Considering these factors, it can be inferred that weight perception, rather than the actual weight itself, is an important predictor of mental health promotion, especially for female adolescents. The seriousness of distorted weight perception lies in its potential to cause inappropriate or unnecessary risks not only in lifestyle choices or weight control strategy selection but also in social relations [19,21,25,30]. The “Thomas theorem,” a sociological argument, has repeatedly emphasized that perception (i.e., weight perception) can be more consequential than objective facts (i.e., BMI), which may lead to significant consequences such as physical and sociopsychological risks [41].

In modern society, the socio-cultural standardized body type ideals tend to favor “slender” or “thin” women and “muscular” men [25,34]. These ideals, set as a social norm, create pressure on individuals, causing them to compare their body type with others and experience greater discrepancies between their actual and perceived body types [13]. The seriousness of the problem is that such a social environment not only induces negative emotions and behaviors toward one’s own body image but also causes stigma and bias against other people’s body image. Female individuals are at a higher risk of experiencing or internalizing weight stigma than male individuals [26]. This is in line with the findings of previous studies, in which even female participants with normal weight or underweight are not free from weight stigma and bias and are forced to have emotional cognitions, such as a negative self-perception or loss of self-esteem [36,42]. Moreover, considering that the prevalence of overweight or obesity in Korea is not as severe as in the western countries [31], the increase in psychological risks such as depression and suicidal tendencies due to overestimation of weight in female adolescents without obesity, revealed in this study, suggests that lookism in Korea is not only prevalent but also exacerbates sex-based imbalance. Therefore, to protect mental health, education on subjective weight perception as well as actual BMI should be provided to all adolescents, and careful attention should be paid to sex-based differences inherent in obesity or weight perception.

### Implications and recommendations

The findings of this study underscore the importance of distinguishing between objective weight status and subjective weight perception in addressing adolescent mental health. The significant associations between distorted weight perception and both depression and suicidality—particularly among female adolescents without obesity—suggest that perceived body image may have a stronger psychological impact than actual BMI. These results highlight the need to incorporate body image perception as a core factor in mental health assessments and interventions.

Furthermore, the observed sex-based differences indicate that mental health strategies for adolescents must be sex-informed and tailored to the differing psychosocial dynamics affecting male and female adolescents. While female adolescents may be more susceptible to internalized distress due to weight overestimation, male adolescents may underrecognize emotional challenges associated with weight underestimation. These findings reinforce the importance of developing comprehensive, sex-specific approaches to adolescent health promotion and suicide prevention efforts.

On a broader level, this study contributes to a growing body of evidence suggesting that mental health interventions in youth populations should move beyond biomedical indicators such as BMI alone and address sociocultural and psychological components, including self-perception and stigma. The integration of such components into school-based programs, clinical screenings, and public health initiatives could help reduce the psychological burden associated with body image distortions during adolescence.

Based on these findings, several practical recommendations can be proposed. First, schools and community health programs should conduct regular screenings for both BMI and discrepancies in weight perception to identify adolescents at heightened psychological risk. Second, mental health programs should incorporate components that address body image concerns, challenge weight-related stigma, and enhance media literacy to counteract unrealistic appearance ideals. Third, sex-specific strategies should be developed and implemented, that acknowledge and respond to the distinct ways in which male and female adolescents internalize body-related pressures. Finally, teacher and school nurse training should be enhanced to improve early identification of at-risk students and ensure timely referrals to appropriate counseling services. These strategies may help mitigate the impact of weight-related psychological stress and contribute to more targeted and effective youth mental health interventions.

### Limitations and future research directions

This study has certain limitations, primarily related to the use of secondary data. First, depression was assessed using a single-item measure, which does not distinguish between depressive symptoms, episodes, or clinical diagnoses, nor does it reflect symptom frequency or severity. Future studies should enhance the validity and reliability of depression assessment by utilizing comprehensive and multidimensional screening tools such as the Patient Health Questionnaire-9.

Second, due to the limitations of secondary data, important contextual variables—such as parental mental health status, family history of weight-related conditions, and the presence of eating disorders—were not available in the KYRBS dataset. Hence, future studies should consider including these variables to strengthen the explanatory framework and address potential intergenerational or environmental influences.

Third, due to the cross-sectional nature of the KYRBS data, our analysis cannot ascertain the temporal sequence or causality between weight-related factors and mental health outcomes. Although statistical methods such as linear regression or structural equation modeling can explore complex relationships between variables, they cannot establish causality without longitudinal data. Therefore, future studies employing longitudinal designs are necessary to clarify the directionality and causal pathways between weight perception, obesity, and mental health in adolescents.

In line with these considerations, the relationship between depression and weight-related factors, including weight perception, may be bidirectional. Depression may lead to changes in appetite and eating behaviors, potentially resulting in underweight or overweight conditions, while negative weight perception may also contribute to depressive symptoms. However, due to the cross-sectional nature of this study, the temporal sequence of these relationships cannot be determined. Longitudinal studies are therefore needed to explore these potential bi-directional pathways and to determine whether these associations differ by sex.

Lastly, although BMI is widely used in population-based health research, it has limitations in accurately representing individual health status. Specifically, BMI does not differentiate between fat and lean body mass, potentially leading to misclassification. Furthermore, the BMI classification system was originally developed based on Western populations, raising concerns about its cross-cultural applicability and relevance to diverse ethnic groups. Future research should consider using more nuanced and individualized measures of body composition that better reflect adolescent health status.

## Conclusions

This study investigated sex-based differences in the associations between weight perception, BMI, and psychological outcomes among Korean adolescents. The findings revealed that distorted weight perception was significantly associated with depression and suicidality, particularly among female adolescents without obesity. In contrast, these associations were not significant among male adolescents without obesity. These findings highlight the distinct psychological mechanisms through which subjective and objective weight factors interact to adolescent mental health.

In light of these findings, it is essential that mental health interventions address not only objective measures such as BMI but also adolescents’ subjective perceptions of their weight. Programs aimed at improving body image awareness, reducing stigma, and providing psychological support should be designed with sensitivity to sex-specific differences. School-based screenings and mental health curricula should incorporate assessments of both actual weight status and body image perception, with particular attention to female adolescents who may be more vulnerable to the internalization of distorted body ideals. The development of sex-informed public health strategies can help promote improved psychological well-being and reduce the risk of depression and suicidality among adolescents.
